# Editorial Notes

**Published:** 1926

**Authors:** 


### Editorial Notes.

---

**Sir George Wills's Resignation.**

It is with very deep regret we learn that Sir George Wills has resigned the Chairmanship of Council of the University of Bristol, a position he held for many years, devoting to it unremitting attention, time and energy. Sir George Wills's services to education are too well known to need mention here: they are only equalled by his princely generosity, permanent memorials of which we have in our University and its kindred institutions. We are glad to know that he still remains a member of Council, and trust he will long be spared to give it the benefit of his advice and experience.

**Editorial Committee.**

Several changes have occurred in the Editorial Committee consequent upon the resignation of Dr. P. Watson Williams (to which we referred in our Summer issue) and that of Mr. J. Lacy Firth, who has been Reporter of the Society's proceedings and in charge of book reviews for many years. His work has been of great service to the *Journal*, and his resignation is much regretted by his colleagues. Dr. J. A. Nixon has been appointed Editor, Dr. Carey Coombs Assistant Editor, Mr. Hey Groves continues as the *Journal's* surgical adviser, and the vacancy on the Committee is filled by Mr. A. E. Iles, who becomes a member *ex officio*

as Treasurer of the Medico-Chirurgical Society, a newly-formed office to which he was elected at the Annual Meeting in October. Henceforward the finances of the Society and the *Journal* will be in the hands of one officer, the Treasurer, instead of being dealt with separately by the Secretary of the Society and the Editorial Secretary. There can be little doubt that this change will be an advantage to the Society as well as to the *Journal*. The arrangement is not entirely new, as for a short time the offices of Secretary and Editorial Secretary were vested in one individual. The increased membership and activity of the Society rendered it impracticable to merge the two offices again, so a Treasurer was appointed, relieving both Secretaries of an arduous part of their duties. The President of the Society (Dr. A. L. Flemming) has consented to continue as Editorial Secretary, a combination of offices held once before by Mr. James Taylor in 1906, but his experience as manager is indispensable at a time when the Editorial Committee has undergone so many changes.

**Queen Alexandra Memorial Hospital, Weston-super-Mare.**

The success of Mr. Henry Butt's efforts to raise sufficient funds to enlarge the Weston - super - Mare Hospital deserves the warmest praise. The foundation stones of the new Queen Alexandra Memorial Building were laid on Armistice Day, and the whole town assisted in the processions and assembly which demonstrated the satisfaction and pride which the town feels at the prospect of possessing a modern and well-equipped hospital commensurate with its growing needs. The cost of the new building is estimated at £40,000, and of this sum £30,000 has already been subscribed in response to the President's persuasive appeals. There can be no question that the remaining £10,000 will be readily forthcoming. Such a development of its hospital was urgently needed at Weston, and we heartily congratulate the town on its achievement.

**Re-opening of the Wellcome Historical Medical Museum.**

ON October 14th the Wellcome Historical Medical Museum, which had been closed for some months for re-arrangement, was re-opened by Sir Humphry Rolleston, Bart. This museum is not so well known as it deserves to be, for it contains an unrivalled collection of rare objects, pictures, sculptures, manuscripts and early printed books illustrating the evolution and practice of medicine and allied sciences from prehistoric times. One of the most interesting sections deals with primitive medicine, surgery and the healing arts amongst savage and semi-civilised peoples of to-day. In another section of the museum are the memorial collections of articles used by or associated with pioneers of medical progress, such as Edward Jenner's original manuscripts and instruments, and a portion of the "Lister Ward" removed from the Glasgow Infirmary. The museum was founded by Mr. Henry S. Wellcome, and is the result of collections made by him during many years, which he offered as the Museum of the Section of History of Medicine to the Seventeenth International Congress of Medicine held in London in 1913.

The Wellcome Museum, which is situated at 54a Wigmore Street, is of immense value to research workers, students and others interested in the subjects with which it deals. Medical practitioners and students are admitted to the Museum without charge. The Conservator, Mr. L. W. G. Malcolm, was formerly Assistant Curator at the Bristol Museum, and his experience as an ethnologist has been of inestimable service in many of the alterations and improvements that have been made, more especially in the Hall of Primitive Medicine, where the gradual rise of scientific thought from folk-lore and magic can be traced in a series of exhibits gathered from all ages.

**Annual Medical Dinner.**

The Annual Dinner of the Bristol Medical School was held on Thursday, December 2nd, in the Victoria Rooms. The chair was taken by Professor E. Fawcett, F.R.S., Dean of the Faculty of Medicine, and the guest of the evening was Professor J. T. Wilson, F.R.S., of Cambridge University. The dinner was attended by 150 past and present students and members of the staff. After the dinner a most successful reception and dance was given by the wives of the staff. This was the first occasion on which the dinner and reception had been held in the Victoria Rooms, now the home of the University Union, and the two festivities have never been more enjoyably combined.

